# Empowering drones in vehicular network through fog computing and blockchain technology

**DOI:** 10.1371/journal.pone.0314420

**Published:** 2025-01-24

**Authors:** Shivani Wadhwa, Divya Gupta, Shalli Rani, Maha Driss, Wadii Boulila

**Affiliations:** 1 Institute of Engineering and Technology, Chitkara University, Punjab, India; 2 Department of Computer Science and Engineering, Chandigarh University, Mohali, India; 3 Robotics and Internet-of-Things Laboratory, Prince Sultan University, Riyadh, Saudi Arabia; 4 RIADI Laboratory, National School of Computer Sciences, University of Manouba, Manouba, Tunisia; Abdul Wali Khan University Mardan, PAKISTAN

## Abstract

The performance of drones, especially for time-sensitive tasks, is critical in various applications. Fog nodes strategically placed near IoT devices serve as computational resources for drones, ensuring quick service responses for deadline-driven tasks. However, the limited battery capacity of drones poses a challenge, necessitating energy-efficient Internet of Drones (IoD) systems. Despite the increasing demand for drone flying automation, there is a significant absence of a comprehensive drone network service architecture tailored for secure and efficient operations of drones. This research paper addresses this gap by proposing a safe, reliable, and real-time drone network service architecture, emphasizing collaboration with fog computing. The contribution includes a systematic architecture design and integration of blockchain technology for secure data storage. Fog computing was introduced for the Drone with Blockchain Technology (FCDBT) model, where drones collaborate to process IoT data efficiently. The proposed algorithm dynamically plans drone trajectories and optimizes computation offloading. Results from simulations demonstrate the effectiveness of the proposed architecture, showcasing reduced average response latency and improved throughput, particularly when accessing resources from fog nodes. Furthermore, the model evaluates blockchain consensus algorithms (PoW, PoS, DAG) and recommends DAG for superior performance in handling IoT data. Fog; Drones; Blockchain; PSO; IoT; Vehicular.

## 1 Introduction

With the progress of Beyond fifth-generation (B5G) networks, the Internet of Things (IoT) has significantly grown in various smart surroundings. This growth has improved people’s daily lives by integrating numerous intelligent devices. Yet, the widespread use of dispersed intelligent IoT devices in smart environments has brought about a range of difficulties with the effective handling of data processing, storage, and transfer [1]. The proliferation of smart gadgets necessitates substantial computational resources, energy consumption, and radio bandwidth, hence necessitating meticulous deliberation and strategic remedies.

The rapid advancement of the IoT has led to the expansion of IoT device usage outside industrial sectors such as smart manufacturing, logistics, and energy trading. These technologies are currently being smoothly incorporated into daily life, exerting an impact on domains such as smart healthcare, cities, and homes [2–5]. In recent years, drones, also known as Unmanned Aerial Vehicles (UAVs), have become widely used IoT devices. This is primarily because they offer exceptional flexibility, adaptability, cost-effectiveness, and compact size [6].

Drones serve as crucial mobile drone edge intelligence by acting as relay stations, enabling the gathering of data from low-power, short-range IoT devices in smart surroundings [7]. This feature not only simplifies the process of collecting data but also enhances energy efficiency optimization in these environments [8, 9]. The unique characteristics of drones, such as their simple deployment, ability to move in three dimensions, and higher probability of establishing direct communication, make them an essential tool for expanding network coverage. This upgrade greatly improves the Quality of Service (QoS) for smart IoT devices while also bringing computing capabilities closer to these devices. Drones essentially serve as dynamic mediators that enhance the connectivity and computing capabilities of IoT devices, creating an ecosystem characterized by enhanced efficiency and service quality [10].

Drones play a pivotal role in the evolution of smart environments within the B5G framework [11, 12]. Operating as intelligent edge nodes, they facilitate data gathering, efficient computing, and localized data model training, making significant contributions to environmental consciousness across various domains [13]. By utilizing drones as intelligent edge nodes, these airborne devices elevate smart environments into hubs of innovation and efficiency. The localized processing and computing enhance overall intelligence while promoting ecological sustainability. Despite their potential, integrating drones into IoT for smart environments presents security and energy consumption challenges. Limited energy resources impact network access lifespan, necessitating a focus on addressing these challenges for sustainable and efficient smart environments. Despite challenges, the integration of drones signifies a stride towards greener and more intelligent technological landscapes [14].

Incorporating drones into IoT networks, where drones assume the role of IoT devices, is commonly known as the Internet of Drones (IoD). This integration has found applications in traffic surveillance, object tracking, and disaster rescue. One primary use of IoD is its sensing service, which involves capturing pictures and videos to gather information about specific locations of interest. Drones subsequently generate various computing tasks, offloading them to the Internet through IoD gateways for further processing. The computed results are then transmitted back to the drones and reported to clients [15]. Typically, drones adhere to predetermined transit routes to visit all designated locations of interest [16].

In numerous applications, drones are enlisted to handle computationally intensive tasks like path planning and pattern recognition. However, the inherent limitations in resources, such as battery power and computing capability, make it challenging for a single drone to efficiently manage complex tasks locally [17]. Consequently, to tackle these demanding computational tasks, researchers have explored the option of offloading computations to a cloud server and retrieving results afterward. This approach significantly augments the capabilities of a swarm of drones virtually, making it well-suited for specific business applications like topographic mapping, resource exploration, and environmental monitoring, where latency and reliability are not critical considerations.

Some practical scenarios often involve computing tasks with stringent low latency and high-reliability requirements, such as object recognition, disaster rescue, and emergency obstacle avoidance. Cloud servers, usually located at a considerable distance from drones, introduce notable latency during data transmission [18]. Meanwhile, in some challenging environments, the absence of a functional wireless infrastructure further exacerbates the issue, rendering cloud-based computation offloading unsuitable for latency- and reliability-sensitive business applications.

Traditionally, drones have relied on offloading computing tasks to remote cloud servers, benefiting from extensive computing resources. However, this approach introduces significant network latency, impacting user QoS. To address this, Fog-aided IoT networks have been proposed, aiming to enhance IoD service performance, especially for time-sensitive tasks. As Fog devices are closer to the drone network, their processing speed also increases. This improves the decision-making power of the drones, whereas higher latency is observed in the case of cloud computing. Fog nodes are placed near IoT devices, typically attached to gateways, providing computational resources for deadline-driven tasks from drones, ensuring swift service responses.

The performance of a drone is notably influenced by its limited battery capacity due to size and weight constraints. Efficient IoD systems must tackle this challenge by minimizing energy consumption. In IoD networks, drone energy usage encompasses wireless communication and propulsion for hovering and transitions [19]. Designing energy-efficient IoD systems requires careful consideration of both types of energy consumption.

To fulfill the increasing demand for drone flying automation, user drones are expected to engage in a broader range of applications, necessitating corresponding network services. Surprisingly, few studies have more focussed on establishing a secure service architecture specifically tailored for drone networks [20–23]. Existing research predominantly employs drones as auxiliary components to address the security challenges of IoD data processing.

The absence of a systematic architecture for drone network services has created a hindrance to the rapid generation of services and applications, consequently hindering the realization of drone flying automation. Recognizing this gap, there is an urgent need for a comprehensive drone network service architecture to catalyze and expedite the implementation of drone flying automation. Consequently, this article addresses this imperative need by proposing a secure, reliable, and real-time drone network service architecture, with a particular emphasis on collaboration with fog computing.

The main contributions of the paper are:

1. Dynamic Trajectory Planning for drones is done on the basis of factors such as velocity, acceleration coefficients, and random values (selection of trajectory path, change in path due to wind, temperature, etc.). The dynamic nature of trajectory planning contributes to efficient and adaptive drone movement within the specified area.
2. Fog Computing for Drone with Blockchain Technology (FCDBT) is proposed for the vehicular network. This architecture is designed to facilitate efficient communication and collaboration among drones, smart vehicles, fog computing nodes, and blockchain technology.
3. Offloading of computation to other fog nodes is done based on the algorithm proposed. This algorithm is based on the Particle Swarm Optimization (PSO).
4. Blockchain technology is used for secure data storage of processed data generated by drones. Different consensus algorithms, including Proof of Work (PoW), Proof of Stake (PoS), and Directed Acyclic Graph (DAG), are used to evaluate the best consensus approach for this data.

The organization of the paper is structured as follows:

Related work of the study is presented in section 2. Section 5 provides the trajectory motion of drones. The system model is formulated in section 4. FCDBT model is proposed in section 3. Section 6 discusses the results. Section 7 concludes the paper.

## 2 Related work

Extensive research has been conducted on ensuring security, managing trajectory control, and optimizing resource utilization for computational tasks.

Authors in [24] thoroughly examined drone security, addressing issues such as collisions within drone swarms, network attacks, and unforeseen problems. They proposed methods to manage task allocation within drone swarms and substantiated the effectiveness of their approach through multiple real physical flying experiments. The research paper [25] endeavors to introduce a conceptual model illustrating the organization of such an architecture and outlines the specific features that an IoD system, based on this architecture, should incorporate to effectively support diverse drone applications. Drones enhance network architectures by positively impacting reliability, connectivity, throughput, and delay. However, challenges such as wireless medium unreliability, battery duration, high mobility, and security and privacy concerns have led to a substantial body of recent literature on IoD-related issues as stated in a recent study [26].

The study [27] examines physical layer aspects in aerial/ground environments, emphasizing channel characterization and modeling through measurement approaches. It classifies models into empirical or analytical categories, detailing empirical studies based on measurement campaigns that consider signal frequency, environmental conditions, and UAV parameters. The article [28] surveys area coverage challenges in UAV networks, addressing factors like coverage capabilities, UAV mobility, network connectivity, and environmental obstacles. Coverage types, categorized by UAV motion and network deployment, are discussed. Energy consumption, UAV coordination, and path planning constraints are briefly outlined, along with adopted path models for studying dynamic coverage problems.

The IoD network relies on the interconnection of drones through the IoT, making it susceptible to security and privacy threats that impact IoT networks. Safeguarding against these threats is crucial for optimal IoD application performance, as security and privacy concerns have considerably limited the broader impact of the IoD paradigm [29]. The study [30] focuses on optimizing multiuser communication scheduling and UAV trajectory to maximize minimum throughput, presenting an efficient iterative algorithm for convergence. Simulation results demonstrate substantial throughput gains compared to benchmark schemes, emphasizing the effectiveness of the proposed design. The research [31] paper explores the role of drones in smart city management, particularly in addressing security concerns. The research emphasizes the integration of AI-based drones to enhance security measures in urban areas. It also discusses the potential contribution of emerging technologies like blockchain to improve overall smart city management. Specifically, both the movement of drones and inadequate security pose potential issues that have not yet materialized. These concerns encompass unauthorized access, increased latency, and elevated energy consumption within the drone network [32]. A novel model-based aggregation technique is introduced for Federated Learning in Industrial Cyber-Physical Systems, enhancing sensor data privacy using Lime for transparency and Blockchain for security. It incorporates transfer learning to improve adaptability to evolving threats and proposes a comprehensive privacy evaluation method [33].

A dynamic collaborative task offloading (DCTO) method is proposed to address challenges in offloading strategies [34]. This method considers fog nodes’ resource status, allowing tasks to be executed by single or multiple fog nodes to reduce latency. Their approach significantly decreased latency, especially under high service request rates, while maintaining low computational complexity for online implementation, distinguishing it from other algorithms. A dynamic programming is utilized to optimize computation offloading for stationary edge nodes [35]. Their novel approach, Dynamic Programming with Hamming distance termination (DPH), addresses the limited battery power of mobile devices. In high network bandwidth scenarios, DPH strategically offloads tasks to the cloud, enhancing execution time and reducing mobile device energy usage. Notably, DPH is scalable and efficiently handles larger offloading problems, showcasing its versatility and effectiveness. Fog computing enhances vehicular ad hoc networks, but load balancing among fog nodes remains a challenge, impacting service availability and resource efficiency. A dynamic resource management (DRM) method is introduced that utilizes service migration to allocate fog resources to autonomous cars [36]. Solved efficiently in polynomial time, the method significantly improves capacity, maintainability, accessibility, availability, and throughput, addressing the load balancing challenge in vehicular ad hoc networks. A task offloading and scheduling method are proposed based on osmosis [37]. This algorithm categorizes devices and tasks and then assigns tasks to nodes based on their respective capacities. The osmotic-based scheduling algorithm exhibited notably superior performance when compared to traditional random and round-robin task offloading algorithms. The effectiveness of this approach was validated through a comparison using synthetic datasets, highlighting its advantages over other algorithms. The existing algorithms either exhibit slow convergence or lack the assurance of reaching the optimal solution. These limitations make them unsuitable for tasks that demand both low latency and high reliability, which is the specific focus of our study.

## 3 FCDBT: Fog computing for drone with blockchain technology

**Fig 1:**
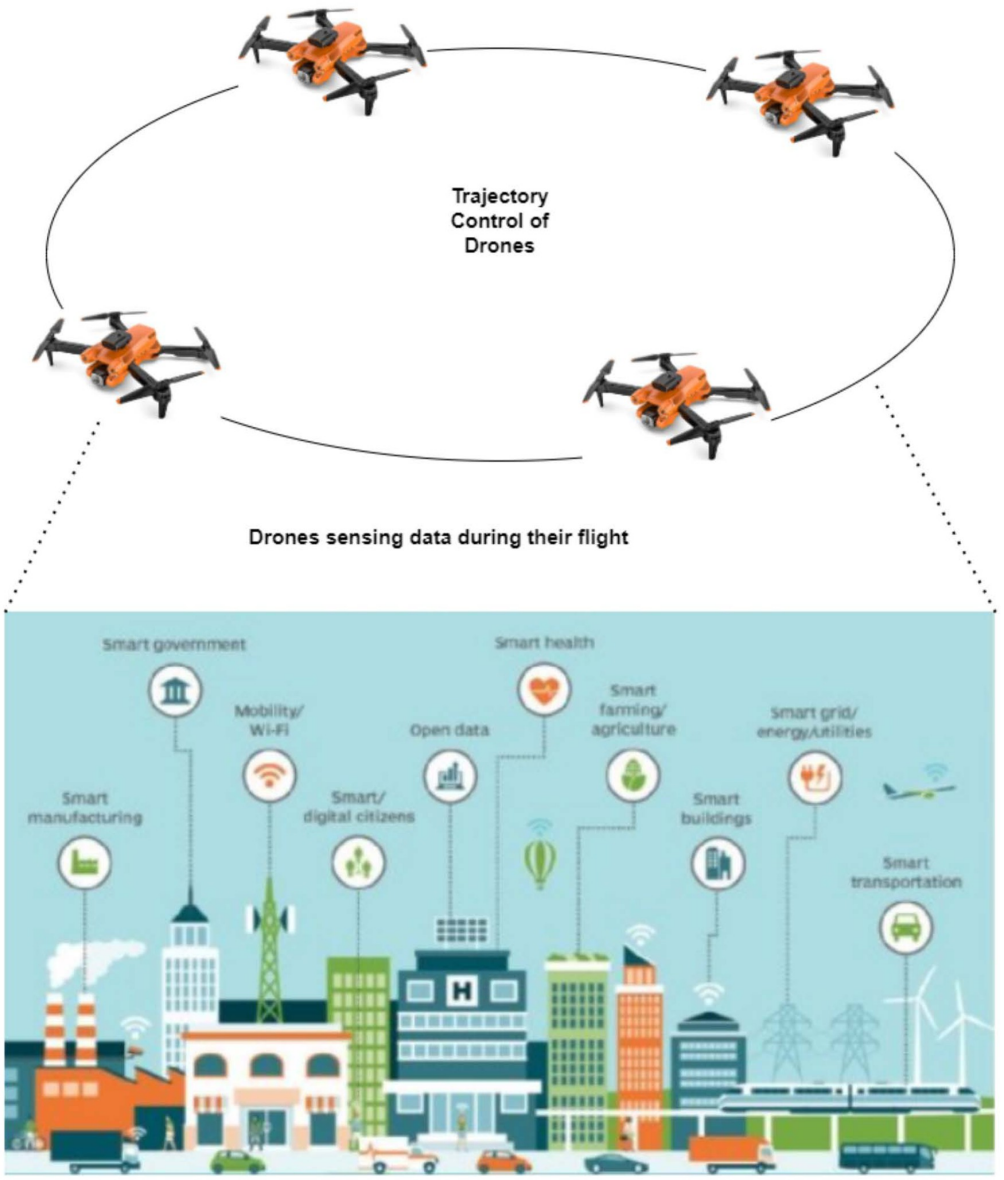
Trajectory area of drones.

The drones encounter a significant demand for computational resources as they engage in the complex task of interpreting data from the IoT during their flight within a designated area. Navigating through this fixed space, the drones actively sense and collect data from the smart vehicular environment, as shown in Fig 1. These IoT devices, which serve as the primary data sources, are inherently constrained by low power and limited computational capabilities. This inherent limitation poses a challenge in directly extracting intelligent data from the vast pool of sensed data.

In response to this challenge, each drone is responsible for autonomously processing a fraction of the overall computation task. However, recognizing the complexity and volume of the data, a collaborative approach is adopted. A portion of the computational task is intelligently offloaded to other drones that traverse within the trajectory area of the originating drone. This cooperative effort transforms the neighboring drones into fog nodes, acting collectively to share and execute computational tasks efficiently. The allocation of the specific fraction of computation to other nodes is intricately determined by a coefficient, a pivotal parameter calculated by the algorithm based on Particle Swarm Optimization (PSO) presented in Algorithm 1. PSO model automatically represents the decentralized networking of drones. Each drone acts as a swarm particle and performs the adjustment on the basis of its own experience and the joint experience of the swarm. This performs the efficient task of offloading and improves trajectory management.

**Fig 2:**
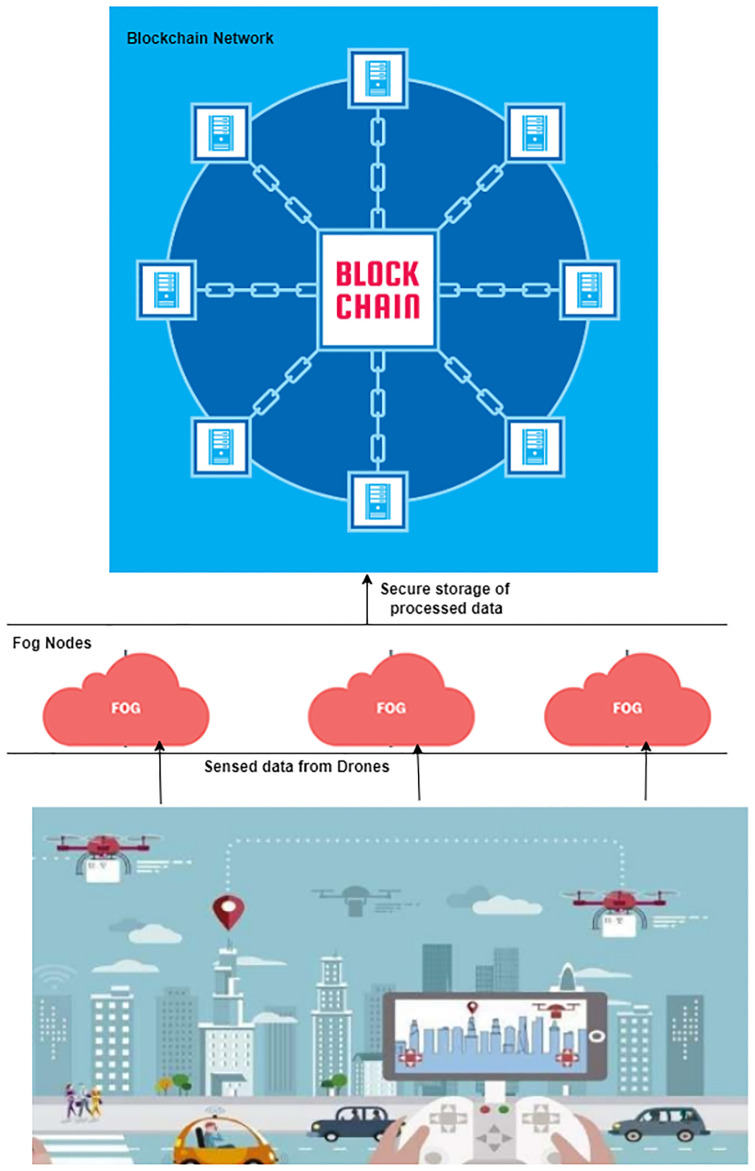
FCDBT model.

A robust solution is implemented to strengthen the integrity and security of the processed data, as shown in Fig 2. The data is securely stored within the blockchain network, a decentralized and tamper-resistant ledger. This blockchain ledger not only safeguards the processed data but also ensures the immutability and confidentiality of the information. This security framework enables the extraction of intelligent decisions from securely processed data, thereby enhancing the overall efficiency and reliability of the drone-based system within the smart environment.

The sensors embedded in drones actively monitor the vehicular network, where intelligent vehicles navigate the roadways. Drones adeptly hover around these smart vehicles, consistently collecting real-time data. Operating at a predetermined altitude and within designated zones, the drones capture and process this information. The processed and securely stored data within the blockchain network plays a pivotal role in facilitating informed decision-making.

Utilizing this processed data, the system provides crucial information to vehicular users. Information regarding impending traffic congestion, accident-prone zones, and other relevant updates is provided to the users in real-time. This proactive approach empowers users by offering alternative routes, enhancing their ability to make informed decisions and navigate traffic intelligently.

**Algorithm 1**: PSO for coefficient computation of offloading

1: Initialize swarms with different combinations of coefficients of computation.

2: Initialize *v*_*i*_ and position *X*(*x*_*i*_, *y*_*i*_, *z*_*i*_) of the *i*th drone moving within the trajectory area.

3:  **while** True **do**

4:   **for** each drone **do**

5:   Compute coefficient, update *pbest*, and *gbest*.

6:   Update inertia weight.

7:   **for** each drone **do**

8:    Update *v* and *X*.

9:   **end for**

10:  **end for**

11: **end while**

12: **return** *gbest* = 0

## 4 System model

‘N’ number of dynamic moving drones are present in the network region of drones. Drones are represented by *d*_0_, *d*_1_, *d*_2_, *d*_3_,……, *d*_*N*_. Due to less computational resources available with the drones, the drone *d*_0_ performs a computational offloading to the nearby drones. Drone, *d*_0_ is moving in its trajectory area. Within its trajectory area, let us assume that n (*n* < *N*) drones are moving.

Number of subtasks, *α* = T/n,

Computation to be done by *d*_0_ = *α* * *β*

Computation to be done by other drones = *α* * (1 − *β*)

Each drone performs computation = *k*_*i*_ * *α* * (1 − *β*)

*k*_*i*_ performs the computation offloading coefficient of each drone present in the trajectory area of *d*_0_.

Velocity of drones,

$$\begin{array}{c} v_{j}\left(t+1\right)=w\cdot v_{j}\left(t\right)+c_{1}\cdot r_{1}\cdot \left(pbest_{j}-X_{j}\left(t\right)\right)+c_{2}\cdot r_{2}\cdot \left(gbest-X_{j}\left(t\right)\right) \end{array}$$ (1)

The velocity variable represented in Eq 1, changing dynamically, determines the speed of the drone in its trajectory motion. By varying the values of w, *c*_1_, *c*_2_, the motion of the drone is affected. w determines the pace with which the coefficient, *k*_*i*_ is computed. A lower value of ‘w’ computes the value of ‘k’ faster.

Effective communication among dynamically moving drones demands data exchange in a synchronized manner. More complexity is introduced in the system when computational tasks are distributed. The drones need to perform in their operational range only, otherwise their exist chances of their failure.

## 5 Trajectory motion of drones

The operator of the drone utilizes a remote controller to transmit signals to the flight controller, specifying the preferred direction and speed for the drone. Advanced flight controller algorithms analyze the inputs received from the remote controller and additional sensors like GPS and a compass. These algorithms then compute the optimal motor speed and direction necessary for executing the specified commands.

### 5.1 Trajectory motion

Deploying a solitary drone for mission execution poses certain challenges. For instance, in reconnaissance missions, a single UAV may encounter limitations in observation angles, restricting its ability to observe the target area comprehensively. When confronted with expansive search tasks, the coverage of a single UAV may prove insufficient for the entire reconnaissance area. Additionally, in offensive operations, constraints on combat range, killing radius, destruction capability, and attack accuracy can hamper the success rate of the overall mission. Furthermore, if a single drone experiences a malfunction mid-mission, an immediate interruption and return are necessary, potentially causing delays and disrupting the entire operational plan in a wartime scenario.

To address these shortcomings and enhance combat effectiveness, proper planning of the dynamic drone’s trajectory is needed. There is a requirement to organize, maintain, or reconstruct a specific geometric formation using multiple drones during task execution. Such formations are designed to adapt dynamically to the ever-changing battlefield situation and the specific requirements of the mission. Dynamic trajectory motion means adapting the flight of the drone based on real-time scenarios of obstacles, climate, sensor data, etc. Dynamic planning will allow the efficient navigation of drones and optimize safety and performance measures.

### 5.2 Trajectory equations

Efficient dynamic trajectory planning is a critical aspect of executing the Internet of Drones. Graphs are instrumental in defining drone targets, encompassing both horizontal and vertical distances. Various algorithms, such as Dubins Curve, Probabilistic Roadmaps, Floyd algorithm [38], Dijkstra algorithm [39], and the Voronoi graph method [40, 41], can be applied to ensure the secure navigation of multiple drones. The dynamic nature of longitude, latitude, and altitude over time characterizes the velocity along the three axes. The trajectory angle and velocity during takeoff determine the drone’s horizontal and vertical distances. The symbols used in the equations are defined in Table 1.

**Table 1:** Symbols used in equations.

Symbol	Meaning
*ϕ*	Latitude
λ	Longitude
*h*	Altitude
*v*_*x*_, *v*_*y*_, *v*_*z*_	Velocities in x, y and z direction
F	Thrust
m	Mass
g	Gravity
*v*_*j*_(*t* + 1)	Velocity of particle *j* at time *t* + 1
*w*	inertia weight
*c*_1_, *c*_2_	Acceleration coefficients
*r*_1_, *r*_2_	Random values (between 0 and 1)
*pbest*_*j*_	Best-known position of drone *j*
*gbest*	Best position in the trajectory area
*X*_*j*_(*t*)	Current position of particle *j* at time *t*

$$\begin{array}{c} \frac{dx}{dt}=v_{x} \end{array}$$ (2)

$$\begin{array}{c} \frac{dy}{dt}=v_{y} \end{array}$$ (3)

$$\begin{array}{c} \frac{dz}{dt}=v_{z} \end{array}$$ (4)

$$\begin{array}{c} \frac{dv_{x}}{dt}=\frac{F}{m}\left(\text{cos}\left(\phi \right)\text{sin}\left(\theta \right)\text{cos}\left(\psi \right)+\text{sin}\left(\phi \right)\text{sin}\left(\psi \right)\right)-g\text{sin}\left(\theta \right), \end{array}$$ (5)

$$\begin{array}{c} \frac{dv_{y}}{dt}=\frac{F}{m}\left(\text{cos}\left(\phi \right)\text{sin}\left(\theta \right)\text{sin}\left(\psi \right)-\text{sin}\left(\phi \right)\text{cos}\left(\psi \right)\right)+g\text{cos}\left(\theta \right)\text{sin}\left(\psi \right) \end{array}$$ (6)

$$\begin{array}{c} \frac{dv_{z}}{dt}=\frac{F}{m}\left(\text{cos}\left(\phi \right)\text{cos}\left(\theta \right)\right)-g\text{cos}\left(\theta \right) \end{array}$$ (7)

$$\begin{array}{c} \frac{d\phi }{dt}=\frac{v_{x}}{h} \end{array}$$ (8)

$$\begin{array}{c} \frac{d\lambda }{dt}=\frac{v_{y}}{\left(h)\text{cos}(\phi \right)} \end{array}$$ (9)

$$\begin{array}{c} \frac{dh}{dt}=v_{z} \end{array}$$ (10)

These equations represent the trajectory position equations of a drone.

Eqs 2–4 represents the motion along three axes, i.e., the x, y, and z axes. Eqs 5–7 describe the adaption of dynamic motion of drone moves in space by keeping in view the impact of thrust (force), gravity, and orientation angles. Eqs 8–10 mention the distance of a drone from the earth’s surface. Here, the curvature of the Earth and gravitational impacts are generally ignored due to the operations of drones on a small scale.

## 6 Results and discussions

The simulation involved ten client threads running on a Win10 Operating System with 8 CPUs and 16G RAM, representing nine user drones. Additionally, specific parameters were pre-set for the application based on real-world drone conditions. The configured maximum flight speed of the user drone is 15 m/s, and the defined trajectory area of the attention area is 90 *m*^2^. The master drone, dr0, is positioned at the origin, denoted as (0, 0, 0). The failure rates for both dr0 and neighboring drones, dri, are assumed to follow a uniform distribution, represented as *v*_0_ and *v*_*i*_ U([0, 0.005]). The open-source traffic simulator SUMO is used to generate the flow of vehicles to simulate the movement trace of vehicles. To benchmark and compare the performance of the proposed model, fog, edge, and cloud resources are used.

### 6.1 Average response latency

**Fig 3:**
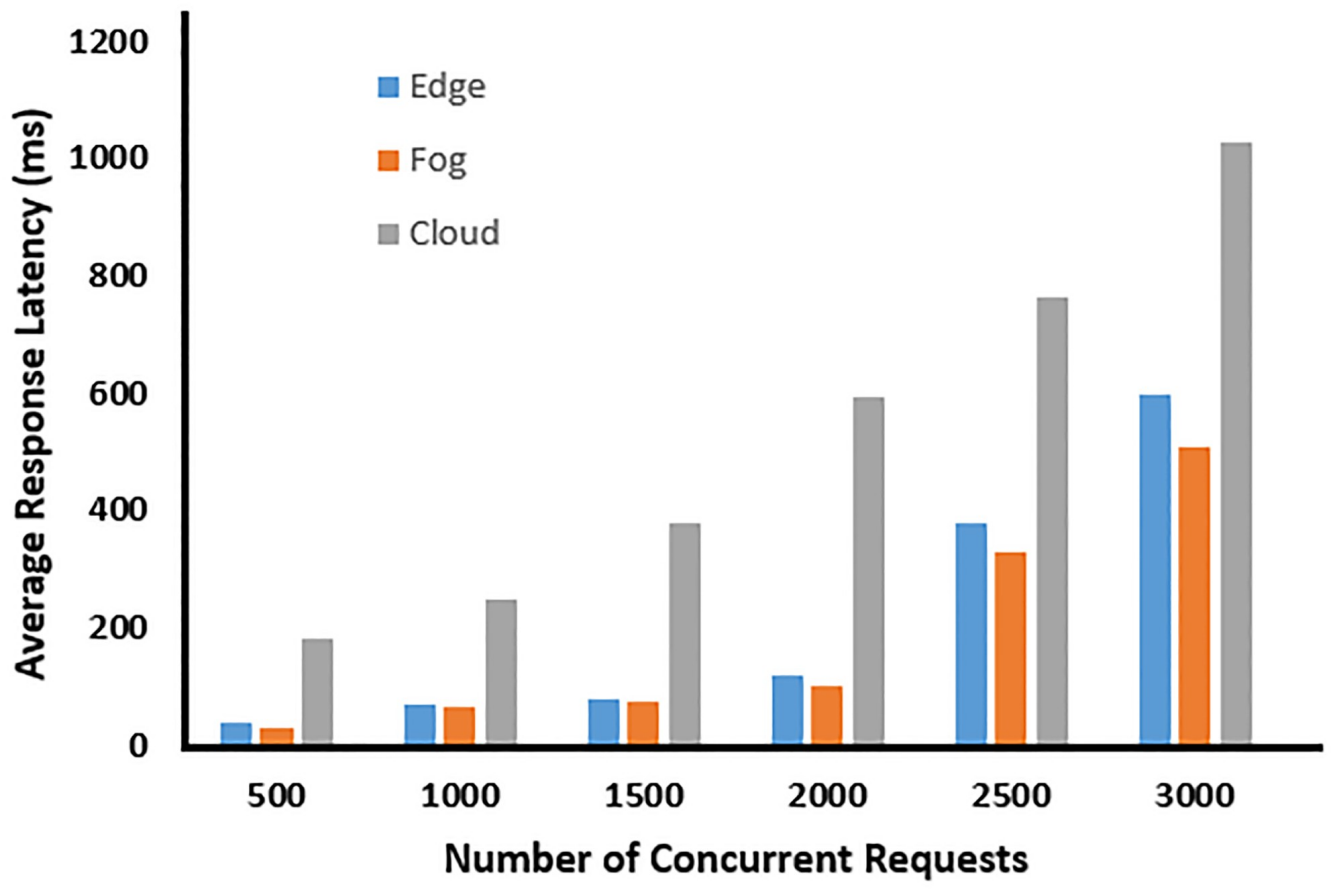
Average response latency.

The average response latency signifies the duration drones require to offload their computations to other devices. Low latency is essential to deliver efficient services to vehicular users, enabling them to make timely decisions. Computation offloading is distributed across fog, edge, and cloud devices, as depicted in Fig 3. With an increase in concurrent requests, drones (fog nodes) situated within the trajectory area respond quickly. This results in reduced latency and enhances the quality of service compared to edge and cloud alternatives.

### 6.2 Throughput in accessing resources from fog, edge, and cloud

**Fig 4:**
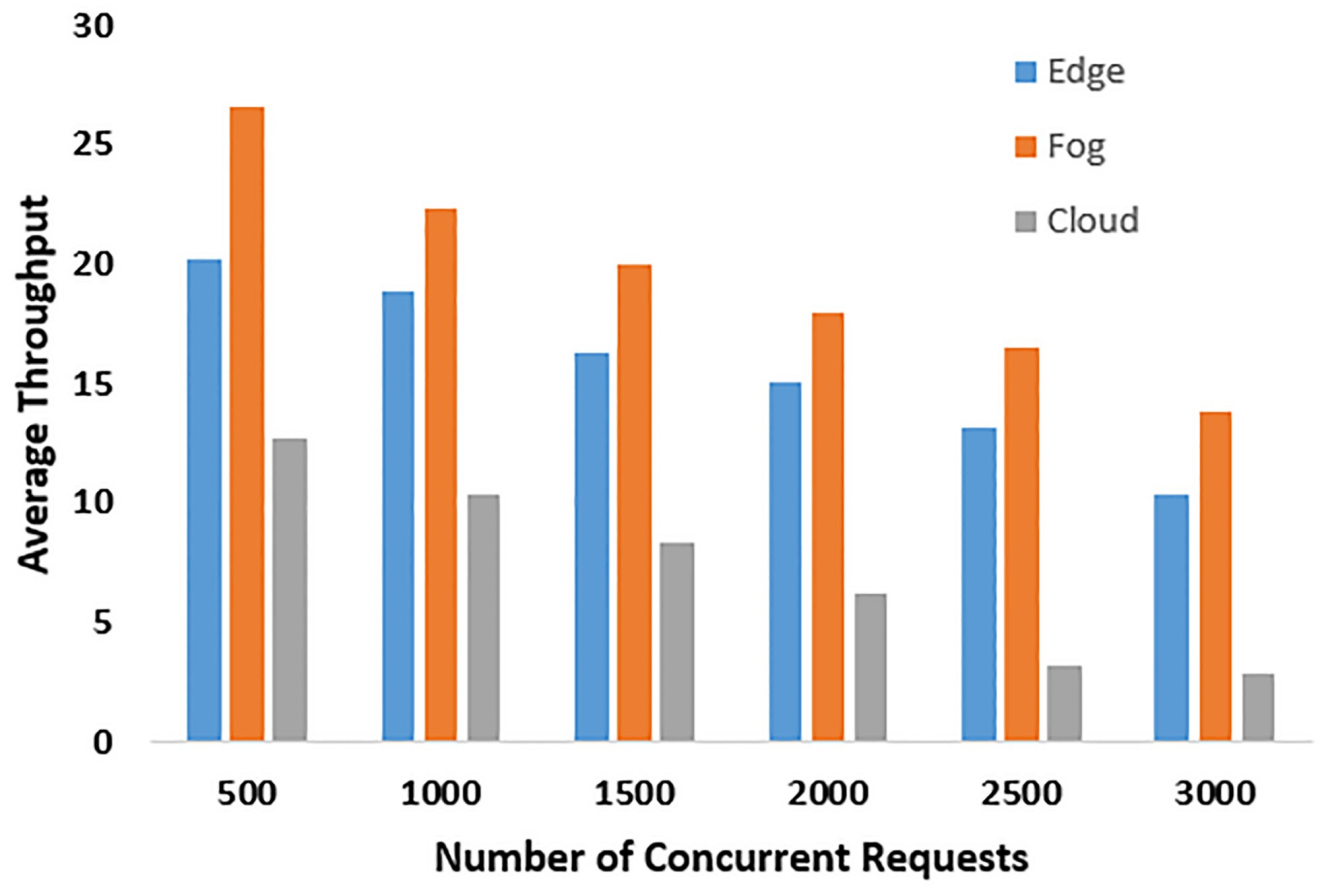
Throughput in accessing resources from fog, edge, and cloud.

The average throughput represents the drone’s ability to handle and process data locally, as well as its efficiency in offloading computations to fog nodes. A comparison was conducted to evaluate the offloading of computations to fog, edge, and cloud nodes, as depicted in Fig 4. Fog nodes exhibit superior throughput primarily because they are readily available within the drone’s trajectory area. The reduced latency at fog nodes contributes to this improved throughput.

### 6.3 Througput in blockchain network

**Fig 5:**
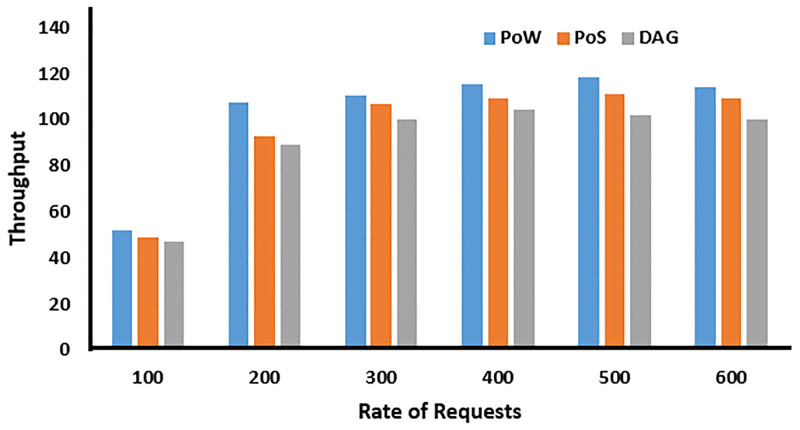
Throughput in blockchain network.

The throughput of a blockchain network is evaluated based on various consensus algorithms employed within the network. This evaluation is conducted using the Blocksim simulator. Consensus algorithms such as PoW, PoS, and DAG are considered in this assessment. DAG demonstrates superior performance, particularly in handling IoT data, when compared to PoW and PoS. The comparison of these three consensus approaches in storing processed drone data is illustrated in Fig 5. DAG’s effectiveness in managing IoT data contributes to its good performance in sensed data. Organization of tasks on the basis of dependencies in DAG helps the drones in processing the critical input data. Hence, latency is minimized, which helps the drones to quickly respond to real-time scenarios.

## 7 Conclusion

This study addresses the escalating need for applications with low-latency and high-throughput capabilities through the widespread adoption of IoT and drone devices. Our work presents the innovative concept of harnessing drones equipped with fog nodes. Trajectory motion and equations are also presented to know the area of motion of drones. The FCDBT model proposed in drone network service architecture addresses security concerns, optimizes computational tasks, and ensures real-time responsiveness. The consideration of limited drone battery capacity is addressed by designing an energy-efficient IoD system. By minimizing energy consumption in both wireless communication and propulsion, the architecture aims to extend the operational lifespan of drones, contributing to sustainable and prolonged use. The integration of blockchain technology ensures secure and tamper-resistant storage of processed data. This not only safeguards the integrity of the information but also provides a decentralized and confidential ledger for storing critical data, enhancing overall security within the IoD system. The proposed model evaluates various consensus algorithms, with DAG emerging as the recommended choice for its superior performance, particularly in handling IoT data. Reduced average response latency, improved throughput, and successful integration of fog computing and blockchain technology demonstrate the architecture’s effectiveness. In the future, this model can be proposed for different types of networks to respond quickly to the decisions made using the processed data of drones.
